# The accurate assessment of small-angle X-ray scattering data

**DOI:** 10.1107/S1399004714010876

**Published:** 2015-01-01

**Authors:** Thomas D. Grant, Joseph R. Luft, Lester G. Carter, Tsutomu Matsui, Thomas M. Weiss, Anne Martel, Edward H. Snell

**Affiliations:** a Hauptman–Woodward Medical Research Institute, 700 Ellicott Street, Buffalo, NY 14203, USA; b Department of Structural Biology, SUNY Buffalo, 700 Ellicott Street, Buffalo, NY 14203, USA; c Stanford Synchrotron Radiation Lightsource, 2575 Sand Hill Road, MS69, Menlo Park, CA 94025, USA

**Keywords:** SAXS data quality, *SAXStats*

## Abstract

Small-angle X-ray scattering (SAXS) has grown in popularity in recent times with the advent of bright synchrotron X-ray sources, powerful computational resources and algorithms enabling the calculation of increasingly complex models. However, the lack of standardized data-quality metrics presents difficulties for the growing user community in accurately assessing the quality of experimental SAXS data. Here, a series of metrics to quantitatively describe SAXS data in an objective manner using statistical evaluations are defined. These metrics are applied to identify the effects of radiation damage, concentration dependence and interparticle interactions on SAXS data from a set of 27 previously described targets for which high-resolution structures have been determined *via* X-ray crystallography or nuclear magnetic resonance (NMR) spectroscopy. The studies show that these metrics are sufficient to characterize SAXS data quality on a small sample set with statistical rigor and sensitivity similar to or better than manual analysis. The development of data-quality analysis strategies such as these initial efforts is needed to enable the accurate and unbiased assessment of SAXS data quality.

## Abstract 2

A set of quantitative techniques is suggested for assessing SAXS data quality. These are applied in the form of a script, *SAXStats*, to a test set of 27 proteins, showing that these techniques are more sensitive than manual assessment of data quality.

## 1. Introduction   

X-ray crystallography and NMR have proven to be highly effective methods to determine the high-resolution structures of many biological macromolecules. However, limitations of each technique have restricted their applicability. This is illustrated using data gathered as part of the Protein Structure Initiative (PSI), which was established in 2000 by the National Institute of General Medical Science in order to determine the structures of a broad range of macromolecules pertaining to biological and biomedical problems. As part of this initiative, large-scale centers were created to establish and optimize structural pipelines. Success and failure, tracked at each stage of the process, showed that ∼12% of soluble purified proteins from the initiative resulted in structures deposited in the Protein Data Bank (PDB; Chen *et al.*, 2004 ▶). Given the nature of the problem this success rate is commendable, but it also illustrates that a large amount of effort was expended to produce the 88% of the samples that to date have provided little to no structural detail.

A complementary technique to X-ray crystallography and NMR is small-angle X-ray scattering (SAXS; Grant *et al.*, 2011 ▶). SAXS is a solution technique that can yield low-resolution structural information about the size, shape and flexibility of a macromolecule (Putnam *et al.*, 2007 ▶). It is virtually unlimited by protein size and can characterize both those samples that do provide structural information and the majority that do not. If SAXS could be performed in a high-throughput manner, it could provide limited structural information on the majority of the samples produced by the PSI, as well as those resulting from other biological investigations that are recalcitrant to crystallographic or NMR approaches.

The ability to perform high-throughput SAXS exists (Blanchet *et al.*, 2012 ▶; Classen *et al.*, 2013 ▶; Perry & Tainer, 2013 ▶), along with the potential to generate vast quantities of data that require analysis. Some semi-automated software is available to quickly provide users with parameters such as the radius of gyration (*R*_g_), the forward scattering intensity [*I*(0)] and the maximum particle dimension (*D*_max_) necessary to enable rapid characterization of SAXS data (Franke *et al.*, 2012 ▶). However, subjective interpretation of data is still required to fully assess the data quality and to ensure that the conclusions that are drawn are not erroneous. While X-ray crystallography and NMR have quantitative standards by which data quality can be assessed, such as minimum *d*-spacing, merging statistics, chemical shift dispersion or peak line width, SAXS has no equivalents of such values.

A set of publication requirements and standards for small-angle scattering data, both X-ray and neutron, has been proposed (Jacques *et al.*, 2012 ▶). These focus on ensuring that the scattering data and any subsequent analysis are presented in a manner such that the interpretations presented can be independently evaluated. However, even with these guidelines, evaluating the raw experimental data is still somewhat subjective and dependent on expertise in the technique. The use of small-angle scattering is growing rapidly in the biological community, and rigorous metrics are needed to assess the initial scattering data in a non-subjective manner. With this in mind, we have built on the proposed requirements and standards to develop metrics that rapidly yield a quantitative assessment of the quality of the initial X-ray scattering data. These are useful for those gaining experience in the technique but also for the rapid evaluation of data in real time, allowing feedback during the experiment. We detail these metrics and the results of their application to previously described SAXS data.

## 2. Experimental   

### 2.1. Macromolecular samples   

Our set of samples consisted of 27 proteins supplied by the Northeast Structural Genomics Consortium (NESG) where a crystallographic structure, an NMR structure or combinations of both were available. The samples are described in detail elsewhere (Grant *et al.*, 2011 ▶). The proteins are representatives from large protein domain families or biomedical themes, or have been selected as targets whose known structure would be significant to the biomedical community (Wunderlich *et al.*, 2004 ▶). Each target is characterized *via* a series of biochemical experiments including analytical gel filtration, static light scattering, mass spectrometry, NMR spectroscopy for determining rotational correlation time and (if possible) high-resolution structural data, and, if diffraction-quality crystals can be formed, X-ray crystallography (Goh *et al.*, 2003 ▶; Bertone *et al.*, 2001 ▶). Most targets are full-length polypeptide chains of shorter than 340 amino acids selected from domain sequence clusters (Liu *et al.*, 2004 ▶; Liu & Rost, 2004 ▶) which are organized in the PEP/CLUP database (Carter *et al.*, 2003 ▶). Each protein cluster corresponds to putative structural domains whose three-dimensional structure is not known nor can it be accurately modeled through homology. The taxa of the targets range from bacteria and archaea to eukaryotes, with a focus on human proteins. Details of the target set are provided in Table 1 ▶.

The initial 27 samples (samples 18 and 19 are identical in the study of Grant *et al.*, 2011 ▶) were obtained from the remainder of the proteins used for crystallization screening by the High-Throughput Crystallization Screening laboratory (HTSlab; Luft *et al.*, 2003 ▶). After the crystallization screening experiments have been set up by the liquid-handling systems, approximately 60 µl of recovered sample remained. While each of these samples has undergone extensive quality control, each undergoes at least two freeze–thaw cycles: first when the protein is shipped to the HTSlab and a second time when the remaining protein is shipped to the synchrotron as described below. For many proteins, a freeze–thaw cycle can be detrimental (Murphy *et al.*, 2013 ▶), causing the protein to aggregate or precipitate. Each protein target was prepared in identical buffer conditions consisting of 100 m*M* NaCl, 0.02%(*w*/*v*) NaN_3_, 5 m*M* DTT, 10 m*M* Tris pH 7.5. This consistency in sample preparation greatly aids efficiency during SAXS data collection.

### 2.2. Data collection   

SAXS data were collected on beamline 4-2 (Smolsky *et al.*, 2007 ▶) of the Stanford Synchrotron Radiation Lightsource (SSRL) utilizing high-throughput data-collection strategies (Martel *et al.*, 2012 ▶). The protein solutions used for SAXS data collection underwent two freeze–thaw cycles as described above with a typical sample volume of 60 µl. The sample was diluted with its matching sample buffer to prepare three solutions using sample-to-buffer dilution ratios of 2:1, 1:2 and 1:5 for each sample. Scattering data from a buffer blank were measured, followed by each of the three concentrations of each sample and a subsequent buffer blank for comparison. A wash cycle took place between each sample concentration. A wavelength of 1.13 Å was used for eight consecutive 2 s exposures collected at each of the three sample concentrations. Solutions were oscillated in a quartz capillary cell during data collection to minimize exposure of the same volume. This series of short exposures is essential in order to reliably identify a signature for radiation damage.

### 2.3. Data analysis   

Scripts were developed and used to statistically test the SAXS data for indications of radiation damage and interparticle interactions; both of these effects can distort SAXS profiles and lead to conclusions that are not experimentally valid. Unwanted trends in SAXS data resulting from radiation or interparticle interactions are measured and the significance of these trends is examined using the linear regression *t*-test described below. Since the detection of radiation damage and interparticle interactions relies on statistical significance, the identification of problematic data is achieved in an objective fashion.

### 2.4. Radiation damage   

SAXS data acquisition from proteins has the potential for an inherent experimental artifact: radiation damage. Ionizing radiation can cause biological macromolecules to form high-molecular-weight oligomers owing to the generation of intermolecular cross-linking reactions, the formation of disulfide bonds or other hydrophobic and electrostatic interactions that lead to tertiary-structural or quaternary-structural changes (Davies & Delsignore, 1987 ▶; Le Maire *et al.*, 1990 ▶). These effects manifest themselves as changes in the Guinier plot, the radius of gyration (*R*_g_), the maximum particle dimension (*D*_max_) and the forward scattering intensity [*I*(0)]. By monitoring these parameters as a function of exposure time, radiation damage can be tracked and evaluated.

#### 2.4.1. Using the linear regression *t*-statistic to evaluate radiation damage   

*R*_g_, *I*(0), *D*_max_ and the similarity of each exposure to the first exposure, χ^2^, are calculated using available software packages described later and a linear regression analysis is used to obtain a *t*-statistic (Kenney & Keeping, 1962 ▶) as a function of the exposure (or the dose received). The *t*-statistic describes the likelihood that a slope is significant. From this we can determine whether or not the trends in SAXS parameters as a function of radiation are significant and therefore whether indications of radiation damage are present. The method of ordinary least squares is used to minimize the sum of the square residuals of the linear regression model. For simple linear regression, the *t*-statistic is given by 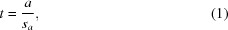
where *a* is the slope of the linear regression, *s_a_* is the standard error in the estimate of the slope, *t* has *n* − 2 degrees of freedom and *n* is the sample size, *i.e.* the number of exposures. The *t*-statistic is converted to a *p*-value to determine the statistical significance of radiation damage, independent of the number of degrees of freedom, using a two-tailed *t*-statistic to *p*-value conversion table (Goulden, 1956 ▶). Radiation damage is evaluated as statistically present if the *p*-value is less than 0.05, a threshold that is commonly chosen to indicate statistical significance. The extent of radiation damage is correlated with the exposure time. Therefore, if the *p*-value is less than 0.05, indicative of damage, then the last exposure is rejected and the fit and *t*-statistic are recalculated for the remaining exposures. This process is repeated until the *p*-value of the linear regression for the remaining exposures is greater than 0.05 or the entire data set is rejected. Exposures that are free from radiation-damage effects in all of the SAXS parameters analyzed are averaged together to improve the signal to noise using the program *DATAVER* (Petoukhov *et al.*, 2012 ▶).

In utilizing the linear regression *t*-test to identify radiation damage, assumptions are inherently made, including the normality of the null hypothesis, the independence of frames and the equal variance of errors. It may be that in specific experimental circumstances these requirements are not met; however, this will generally lead to an inflation of false positives, *i.e.* that frames are identified as damaged when they are not. This will result in fewer exposures averaged and therefore to a decrease in the signal to noise of the averaged profile. Thus, this is an overly cautious test to ensure that radiation-damaged exposures are not included in the final analysis.

#### 2.4.2. Detecting overall changes in the scattering profile   

Typical scattering profiles cover structural information ranging from resolutions of hundreds of Å to as high as 10 Å. Two scattering profiles can be directly compared for overall similarity using the reduced χ^2^ statistic employed in the program *DATCMP* (Petoukhov *et al.*, 2012 ▶) defined as
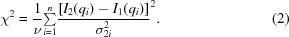
Here, *n* is the number of data points *i*, *I*_1_(*q*_*i*_) and *I*_2_(*q*_*i*_) are the intensities of the scattering profiles of interest at *q_i_* with error σ_*i*_ and *v* is the number of degrees of freedom. For two identical scattering profiles χ^2^ = 0, while two similar profiles will have χ^2^ approximately equal to 1 and two dissimilar profiles will have χ^2^ much greater than 1. While in general the comparison of two scattering profiles is a nontrivial task, this simple discrepancy criterion can be used because we are comparing multiple exposures of the same sample, which will be similar in both scale and noise.

Each scattering profile for subsequent exposures is compared against the first exposure and the χ^2^ is calculated. The first exposure is used for comparison since it received the lowest dose of ionizing radiation. Exposures showing evidence of radiation damage based on χ^2^ are identified and removed from averaging.

#### 2.4.3. Detecting changes in maximum particle dimension   

Radiation exposure can alter the surface and structural properties of proteins as well as solution properties affecting interparticle interactions, all of which may influence the effective maximum particle dimension (*D*_max_) of a protein. SAXS data can be used to estimate *D*_max_ from the pair distribution function [*P*(*r*)] using the program *GNOM* (Semenyuk & Svergun, 1991 ▶). A Fourier transform is used to evaluate *P*(*r*) from *I*(*q*) according to the equation
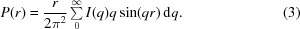



 In practice data cannot be collected from zero to infinity and an indirect Fourier transform method is instead used (Glatter, 1977 ▶) with *D*_max_ selected such that the resulting *P*(*r*) decays smoothly to zero without significant oscillations or systematic deviations in the curve. Typically, a predicted *D*_max_ of 3.5*R*_g_ is used as a starting point and the value is increased or decreased until a suitable value is identified. In addition to calculating *P*(*r*), *GNOM* also calculates the inverse Fourier transform to analyze how well the resulting *I*(*q*) fits the scattering profile. This is an important step to determine the most accurate values of *D*_max_ and *P*(*r*). In the program *DATGNOM* (Petoukhov *et al.*, 2012 ▶) this process has been automated and a series of perceptual criteria such as oscillation, stability and deviation of the fitted *I*(*q*) *versus* the experimental *I*(*q*) are used to select the optimal *D*_max_. Exposures showing evidence of radiation damage based on their *D*_max_ are identified and removed from averaging.

#### 2.4.4. Detecting changes in *R*_g_ and *I*(0)   

Increases in the average size of particles in solution can increase the measured *R*_g_ and *I*(0). *R*_g_ and *I*(0) are examined as a function of exposure number. *R*_g_ can be calculated from scattering data using two independent methods: a Guinier plot analysis and *via* the pair distribution function *P*(*r*). The first method calculates *R*_g_ from the Guinier plot. For a monodisperse solution of globular particles, the Guinier plot ln*I*(*q*) *versus* *q* ^2^ is linear in the low-resolution regime where *q* < 1.3/*R*_g_ (Guinier & Foumet, 1955 ▶). In this region the slope of the line passing through the data is related to *R*_g_,
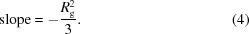



The value of *R*_g_ calculated from the Guinier region varies with user interpretation and expertise. *R*_g_ is calculated from the slope of the line through the data points in the Guinier region. The Guinier region is dependent upon the *R*_g_. The value of *R*_g_ is typically determined through an iterative cycle of calculating *R*_g_ and adjusting the Guinier region accordingly, followed by recalculating the *R*_g_, for several cycles. The final Guinier region and the calculated value of *R*_g_ are determined through a somewhat subjective interpretation of what is an acceptable linear region. This becomes particularly difficult when particles are large, resulting in very few data points and a heightened sensitivity to the estimation of *R*_g_ when varying the Guinier region by as little as one data point. This procedure is automated in the program *AutoRg* (Petoukhov *et al.*, 2012 ▶) which determines the Guinier region by fitting several slightly different regions, calculating the *R*_g_ for each region and evaluating how the *R*_g_ changes as a function of additional data points, and then accepts the region that minimizes the variance in *R*_g_. Cases that contain few points in the Guinier region as a result of a large *R*_g_ or data that are particularly noisy are inherently difficult to calculate using this approach. Methods to utilize non-ideal data with a high success rate are desirable, but it is critical to ensure that the Guinier region and *R*_g_ are determined accurately.

To minimize subjectivity in estimating the Guinier region, we employ an independent method to determine the *R*_g_. In the previous section we described the determination of the maximum particle dimension and the pair distribution function calculated using *DATGNOM*. Using this *P*(*r*) one can calculate the *R*_g_ of the particle from
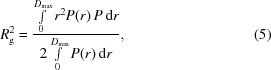
where *D*_max_ is the maximum particle dimension, *r* is the interatomic distance and *P*(*r*) is the pair distribution function (Putnam *et al.*, 2007 ▶). While the determination of *P*(*r*) still requires an estimation of *D*_max_, there is an advantage to calculating *R*_g_ using this method. In the Guinier approximation, even slight modifications to the Guinier region by as little as a few data points can result in a significantly different *R*_g_. Using (5), the *R*_g_ is estimated from the pair distribution function, which in turn has been calculated using all available data points in *I*(*q*), greatly exceeding the number of data points in a Guinier plot and incorporating information from all regions of reciprocal space. While small errors in the estimation of *D*_max_ may alter *P*(*r*) slightly, they have little effect on integration over all *r*, thus providing a robust calculation of *R*_g_ (Jacques & Trewhella, 2010 ▶). Once we have determined the *R*_g_ from this method, we now use this *R*_g_ to determine the Guinier region.

Until now, we have considered the upper limit of the Guinier region to be 1.3/*R*_g_. Occasionally, data at very low resolution close to the beam stop can be influenced by external factors such as parasitic scatter and divergence in the beam (Wignall *et al.*, 1990 ▶; Li *et al.*, 2012 ▶). To alleviate the adverse effects of such factors, it is advantageous to select a minimum cutoff for *q* such that the desired information about shape and size is not lost or distorted. The minimum *q* value required to accurately restore the size and shape information present in the form factor (see §2.5.1) is given by the Shannon sampling theorem, which states that the information content in the continuous function *I*(*q*) can be represented by its values on a discrete set of points termed Shannon channels (Svergun & Koch, 2003 ▶). A measure of the information content is given by Shannon’s sampling theorem, such that
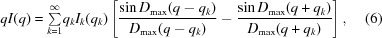
where *q_k_* = *k*π/*D*_max_. The number of parameters required to represent *I*(*q*) over an interval (*q*_min_, *q*_max_) is given by the number of Shannon channels 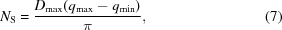
where *q*_max_ and *q*_min_ here refer to the highest and lowest resolutions collected in the experiment, respectively. This provides a lower bound on *q*_min_ such that its value does not exceed the first Shannon channel, *i.e.* that
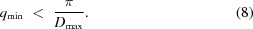



By utilizing this boundary as the lower limit of the Guinier region, *q*_min,G_, and our previously described upper boundary of *q*_max,G_ = 1.3/*R*_g_, we limit the Guinier region to the interval
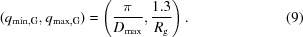



After selecting the data points in this Guinier region, we calculate *R*_g_ using the Guinier method. This second, independent measure of *R*_g_ is compared against the value estimated from *P*(*r*) (Putnam *et al.*, 2007 ▶). If the *R*_g_ from the Guinier approximation differs significantly from the *R*_g_ calculated using the pair distribution function, then there may be additional interparticle interactions affecting only the low-resolution data.

In addition to the slope of the fit to the Guinier region, we can also obtain the scattering intensity extrapolated to *q* = 0. This extrapolated intensity, *I*(0), is directly proportional to the square of the number of electrons in the particle, *i.e.* the molecular weight (Putnam *et al.*, 2007 ▶). The *R*_g_ and *I*(0) are subsequently analyzed and the linear regression *t*-statistic is used to reject data that show changes as a function of exposure indicative of potential radiation damage.

### 2.5. Interparticle interactions   

#### 2.5.1. Concentration dependence   

The intensity data collected by SAXS experiments are given by
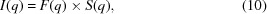
where *F* is the form factor of the particle and *S* is the structure factor. Ideally, particles in dilute solution conditions act independently of one another, exhibiting no interparticle effects, resulting in a structure factor of unity. X-ray exposure time increases signal to noise at the expense of radiation damage. Another variable that can be used to increase the signal to noise is the protein concentration. However, as the concentration of the particle in solution is increased, the average distance between individual particles decreases, and therefore the likelihood of their interaction increases. Variations in electrostatic charge or hydrophobic regions distributed across the surface of the particle can result in either attractive or repulsive forces between neighboring particles with increased concentration, effects that are dependent on the surrounding chemical environment. These interparticle interactions directly affect the structure factor in (10), causing it to deviate from unity. This results in a breakdown of the assumption that *I*(*q*) collected in a SAXS experiment can be treated as the form factor, which contains the size and shape information desired. It is thus important that no interparticle interactions are present in the course of a SAXS experiment.

We use a method similar to the evaluation of multiple exposures as a function of radiation dose to monitor for the presence of interparticle interactions, evaluating the SAXS-derived parameters as a function of concentration. A minimum of three concentrations is required for linear regression analysis. If the *t*-statistic shows a significant trend, this suggests that interparticle interactions exist and are correlated with sample concentration. Interparticle inter­actions may be alleviated by lowering the sample concentration or by modifying the solution conditions.

If very slight levels of interparticle interactions are detected, then either the data can be merged from high and low concentrations or zero extrapolation can be used to generate a curve with sufficient signal to noise while alleviating the influence of interparticle interactions (Petoukhov *et al.*, 2012 ▶).

#### 2.5.2. Scaling SAXS profiles   

An important step in assessing the linear regression for a series of data points is determining the independent variable. In the case of testing for concentration dependence, this step is nontrivial. The independent variable is no longer the exposure number but is the concentration of the particle in solution. Multiple concentrations of a sample are typically prepared either by performing serial dilutions, which aids in creating a linearly dependent set of concentrations, or individually. However, in practice it is often the case that errors in estimated concentration occur. Examples of this include inaccurate initial protein concentration values, the standard deviation associated with dispensing microlitre volumes of liquid and effects such as solvent evaporation over time. All of these can contribute to unpredictable changes in the concentration. To some extent this can be alleviated through the use of in-line UV spectroscopy; however, this is not always available.

To determine an appropriate abscissa for each concentration, we evaluate each concentration in a sample series on a relative scale, *i.e.* one that is not dependent on knowing the absolute solution concentration in mg ml^−1^. However, this is a nontrivial task. Simply dividing the *I*(0) of each concentration by the *I*(0) of the lowest concentration has the flaw that *I*(0) is also dependent upon the molecular weight of the particle in solution, and if interparticle interactions are occurring as a result of increasing concentration then the relative scaling factors will be skewed. Scaling the scattering profiles using the full *q* range is likely to be more accurate but also suffers from increased noise and buffer-subtraction inaccuracies that become more pronounced at higher resolutions.

We select from the data a region of 50 data points beginning at *q* = 0.07 Å^−1^ as this region (in the case of the experiments described here) should not experience distortion from small changes in interparticle interactions for most proteins while still being at a low enough resolution to avoid the region most sensitive to low signal to noise. Each data point in the scaling region for each concentration is divided by the corresponding data point in the first concentration, yielding a list of ratios. This list is sorted from least to greatest and the median ratio is selected as the scale factor for that concentration. Lastly, the first concentration is given an abscissa of 1, while each additional concentration is given an abscissa equal to its corresponding scale factor. These abscissae are now used as the regressors in the linear regression analysis to detect changes as a function of concentration.

#### 2.5.3. Detecting changes in *R*_g_, *D*_max_ and *I*(0)   

Interparticle interactions or changes in particle size as a function of concentration may manifest as changes in *R*_g_ and *D*_max_. Firstly, the pair distribution function is used to calculate *R*_g_ and *D*_max_ using *DATGNOM*. These values are subsequently used to determine the Guinier region, from which (4) is used to calculate *R*_g_ according to the Guinier approximation after using the least-squares method to best fit the data. Three different Guinier regions are used to calculate *R*_g_. All three Guinier regions terminate with a maximum value of *q*_max,G_ = 1.3/*R*_g_, where *R*_g_ has been calculated from the *P*(*r*) distribution. However, each of the three regions utilizes different minimum *q* values. The first Guinier region uses all available data points less than *q* = 1.3/*R*_g_. However, as mentioned above, data points at the lowest *q* values closest to the beam stop may suffer from parasitic scatter. Another Guinier region, previously described by (9), uses the maximum particle dimension to determine the minimum *q* value necessary to reconstruct the continuous *I*(*q*) function from a discrete set of points, and is defined as *q*_min,G_ = π/*D*_max_. However, a potential drawback of using this Guinier region is that depending on the particle size and shape this region may contain very few data points, making it more susceptible to inaccuracies in *R*_g_ calculation owing to noise. To alleviate the potential problems associated with both Guinier regions, we have utilized an additional Guinier region that may ensure the most accurate Guinier estimates in these cases. This region is defined by *q*_min,G_ = 0.65/*R*_g_, *i.e.* half of *q*_max,G_, which for most particle sizes and experimental setups falls between the minimum *q* value collected and the theoretical minimum *q* value required by information theory. To summarize, the three Guinier regions evaluated are
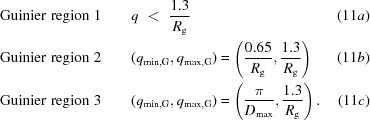



Interparticle interactions may also result in changes in *I*(0), which is proportional to the square of the number of electrons in a particle and hence to the molecular weight. Similarly to *R*_g_, *I*(0) can also be determined from the real-space *P*(*r*) function according to
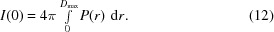



For each concentration (after scaling) the *I*(0) is calculated using both (12) and the *y* intercept of the linear regression in the Guinier approximation. In total, four *R*_g_ values and four *I*(0) values are calculated [one from the real-space *P*(*r*) and one from each of the three Guinier regions]. These eight values, along with *D*_max_, are each analyzed as a function of concentration (using the scale factors described in the previous section) and tested for concentration dependence using the linear regression *t*-test. The resulting likelihood of dependence is expressed as a *p*-value and reported.

#### 2.5.4. Detecting changes in particle volume   

Occasionally, owing to the shape of a particle, increases in particle size may not significantly or detectably alter *R*_g_ or *D*_max_. Another measure of particle size is the particle volume. This value can be calculated from a SAXS profile and is based on the observation by Porod that globular particles that have a sharp interface between the surface and the solvent display a decay in intensity in the high-resolution region proportional to *q* ^−4^ (Porod, 1951 ▶). Porod found that the volume of the particle could be calculated according to the equation
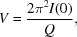
where *V* is the Porod volume and *Q* is the Porod invariant such that
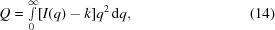
where *k* is a constant subtracted to ensure that the asymptotical intensity decays proportional to *q* ^−4^. Experimentally, one cannot collect data from zero to infinity and instead *Q* is estimated from the convergence at high *q* values. This calculation is provided by the program *DATPOROD* (Petoukhov *et al.*, 2012 ▶) and requires the output from *DATGNOM*, described in the previous section. The Porod volume is directly proportional to the molecular weight of the protein and can be estimated assuming a typical density of 1.37 g cm^−3^ for globular particles according to
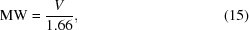
where MW is the molecular weight in daltons (Rambo & Tainer, 2011 ▶). The molecular weight is tested for concentration dependence, and can be compared with the predicted molecular weight for consistency and to check for the presence of oligomers.

#### 2.5.5. Estimating concentration   

Determining the likelihood of concentration dependence does not require knowledge of the absolute concentration; however, calculating the impact of concentration dependence, *i.e.* the slope of the regression, does. To quantify the degree of concentration effects, we estimate the concentration of each sample directly from the data. To obtain an estimate of the concentration of a sample, calibration can be performed using the forward scattering of water or of a model protein of known concentration such as lysozyme or glucose isomerase. These methods are accurate to ∼10% (Mylonas & Svergun, 2007 ▶; Orthaber *et al.*, 2000 ▶). These data, along with *I*(0) calculated from the *P*(*r*) distribution and the scaling procedure described above, allow an estimation of the absolute concentration required to quantify the impact of concentration dependence. The data described here were calibrated using water scattering as the standard.

### 2.6. Evaluating linearity in the Guinier region   

In discussing radiation-damage indicators, we described in detail the method for properly estimating the Guinier region for calculating *R*_g_. While this is an effective method, it does so regardless of the linearity of the data in the Guinier region. Linearity in the Guinier region is an important prerequisite to ensure monodispersity (Jacques & Trewhella, 2010 ▶). If the data here are nonlinear, this suggests that interparticle interactions or aggregation are present in the sample.

**Figure 1:**
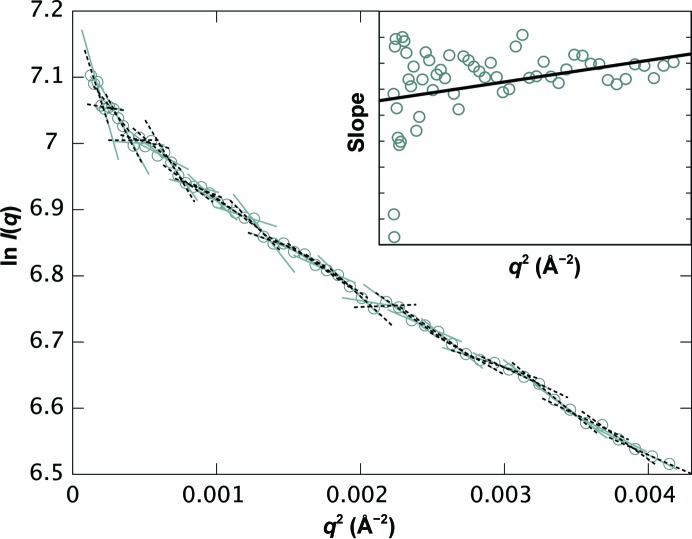
Detecting nonlinearity in Guinier plots. A typical example of a Guinier plot for Guinier region 1 is shown. Data points are plotted as gray circles. The linear fit through each set of three data points is plotted with alternating solid gray and dashed black lines for clarity. A plot of the slope of each fit is shown in the inset. The set of slopes is fitted with a linear regression, shown by the solid black line. Guinier regions that are linear will show a flat line with no dependence on *q* ^2^. Guinier regions that are nonlinear will exhibit a dependence on *q* ^2^, detected using the *t*-­statistic described in the text. This process is performed for each of the three Guinier regions.

To supplement our current analysis, we evaluate whether or not the data in the Guinier region are linear for each concentration. After determining the three intervals for the Guinier region, the method of least squares is applied to fit each block of three consecutive data points, the minimum required to calculate a linear regression. The slope of the line through these three points is calculated, the block of points is shifted by one data point and the procedure is repeated (Fig. 1 ▶). Next, a linear regression is calculated for the set of slopes, *i.e.* the slope of the slopes (Fig. 1 ▶, inset). If the region is linear then the slope of each consecutive block of three points should be constant and independent of the location of the block in the Guinier region. Using the *t*-statistic, we calculate a *p*-value for the likelihood that the trend is significant and therefore that the Guinier region is nonlinear. The slope of the linear regression is used to determine whether the interparticle interactions are attractive or repulsive. If the slope of the regression is positive, this suggests that the interactions are attractive. If the slope of the regression is negative, the interactions are repulsive.

### 2.7. Robustness   

To help to enable the success of the analysis on a wide range of data quality, we have included an optional mechanism for outlier detection. *R*_g_ estimated from the pair distribution function is the parameter used to detect outliers, since it is one of the most robustly determined parameters (Jacques & Trewhella, 2010 ▶). We have utilized the modified *z*-score methodology (NIST/SEMATECH e-Handbook of Statistical Methods, 2012 ▶) of outlier detection with a cutoff of 3.5. This method is particularly robust with small sample sizes as it uses the median of the distribution instead of the mean, and is therefore less likely to be skewed by outliers. In the present study we have enabled outlier detection for radiation-damage analysis only, and have disabled it for the analysis of multiple concentrations since all three concentrations are required to assess concentration dependence.

### 2.8. Scripts   

The basic data-analysis steps described have been coded into a script called *SAXStats*, which makes use of many of the programs provided in the *ATSAS* package (Petoukhov *et al.*, 2012 ▶). *SAXStats* has been written in shell language and, although designed around the protocols and data format output by beamline 4-2 at SSRL, is readily available from the authors for adoption, adaptation or the production of a more generally applicable version. This has been applied to the data described in §[Sec sec2.2]2.2 and the results are described below.

## 3. Results   

**Figure 2:**
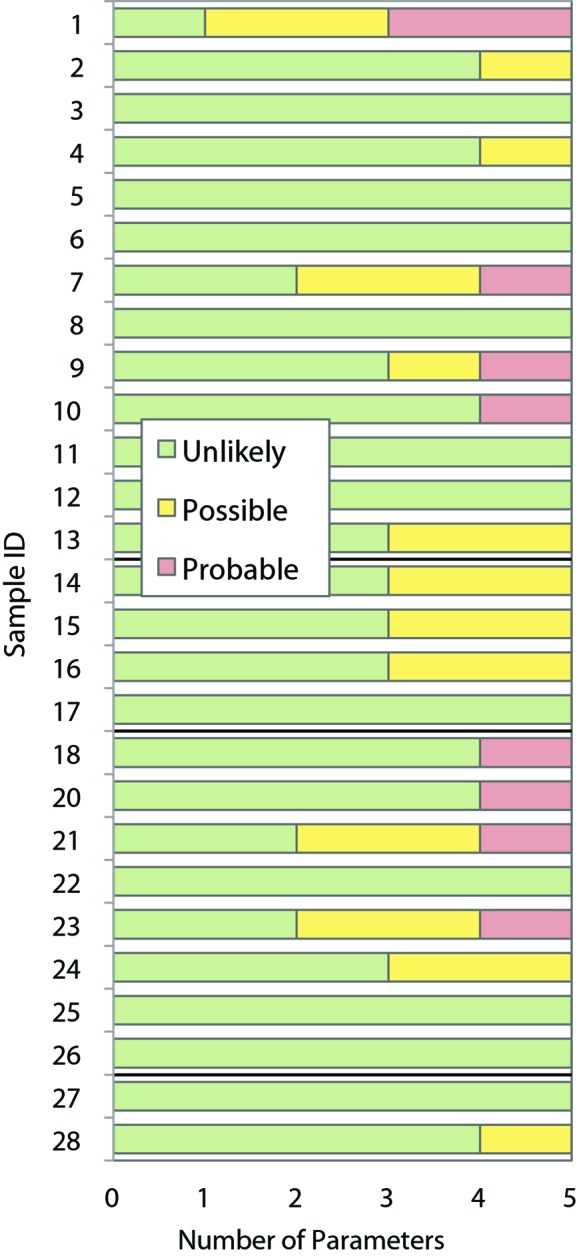
Correlation frequency plot for radiation-damage analysis for the highest concentration of each sample. The number of SAXS parameters (out of five in total) that were unlikely to be (green, *p* > 0.20), possibly (yellow, 0.05 < *p* ≤ 0.20) or probably (red, *p* ≤ 0.05) affected by radiation damage is shown. Any exposures that were affected by radiation damage (*p* < 0.05) in any of the five parameters analyzed were rejected from averaging (Supplementary Table S1). Thick black lines delineate between the three categories of samples described in Table 1 ▶.

**Figure 3:**
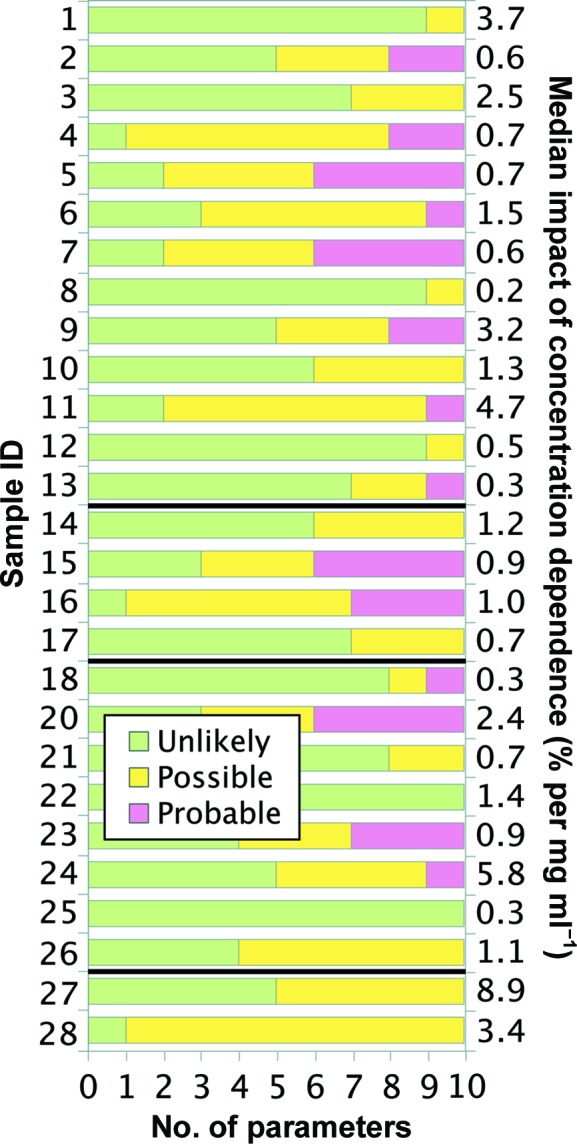
Correlation frequency plot for concentration-dependence analysis. The number of SAXS parameters (out of ten in total) that were unlikely to be (green, *p* > 0.20), possibly (yellow, 0.05 < *p* ≤ 0.20) or probably (red, *p* ≤ 0.05) affected by concentration dependence is shown. For each sample, the absolute value of the slope of the linear regression for each of the ten parameters has been calculated as a percentage of the *y* intercept of the regression. The median of these values is shown to the right of the chart to describe the typical impact that the concentration dependence has on the determination of SAXS parameters for each sample. Thick black lines delineate between the three categories of samples described in Table 1 ▶.

For all 27 samples studied, *SAXStats* successfully calculated all parameters and analyzed each sample for radiation damage and concentration dependence. To rapidly visualize the results of the analysis we introduce a new plot, termed the correlation frequency plot, to describe graphically the information presented in tabular form in the Supporting Information. Here, the sample ID is on the vertical axis, while the number of parameters with a given *p*-value is shown on the horizontal axis. The likelihood of a correlation being present is determined by the *p*-value, which is identified by color as unlikely (green, *p* > 0.20), possible (yellow, 0.05 < *p* ≤ 0.20) or probable (red, *p* ≤ 0.05). For example, in Fig. 2 ▶ sample 1 had one parameter identified as unlikely to be affected by radiation damage, two parameters identified as possibly damaged and two parameters that are probably affected by radiation damage. The specific information about which parameters are affected can be found in the Supporting Information. For the concentration-dependence analysis, the degree of correlation is presented alongside the graph as the median impact of concentration dependence in units of percent per mg ml^−1^. For each parameter, the impact of the concentration dependence is given by the slope of the regression, and the median impact for all parameters collectively is then calculated. For example, in Fig. 3 ▶ sample 1 shows a median impact of concentration dependence of 3.7% per mg ml^−1^ of solution, meaning that each parameter changed by approximately 3.7% for every mg ml^−1^ increase in concentration; however, since nearly all of the parameters are green, the impact of each was determined to be insignificant given the standard errors in the parameters.

### 3.1. Radiation-damage analysis   

The likelihood of radiation damage affecting all five parameters analyzed [*i.e.* *R*_g_ *via* Guinier, *R*_g_ *via* *P*(*r*), *I*(0), *D*_max_ and the similarity of each exposure to the first exposure, χ^2^] for all eight exposures for each of the protein samples is shown in the correlation frequency plot, *i.e.* a plot showing the total number of parameters for which a correlation has been found between the parameter and exposure number (Fig. 2 ▶). For brevity, only the highest concentration is shown. While several parameters analyzed do appear to be affected by radiation damage (Supplementary Table S1), in nearly all cases the effect averaged less than 1% (Supplementary Table S2), demonstrating that the high-throughput experimental protocol is sufficient for collecting data while minimizing radiation damage and that most samples could endure even more exposure before experiencing deleterious effects caused by radiation damage.

### 3.2. Interparticle interactions   

The results from the concentration-series analysis for all samples are shown in a correlation frequency plot in Fig. 3 ▶ and Supplementary Tables S3 and S4. For many samples some parameters are affected by concentration dependence; the impact of this effect is usually less than 5% per mg ml^−1^. However, in cases such as samples 24 and 27 not only is concentration dependence detected in multiple parameters, but the effect is large relative to other samples, showing an impact of 5.8 and 8.9% per mg ml^−1^, respectively. However, this dependence did not appear to affect modeling as shown by the agreement with high-resolution structural data (Grant *et al.*, 2011 ▶).

**Figure 4:**
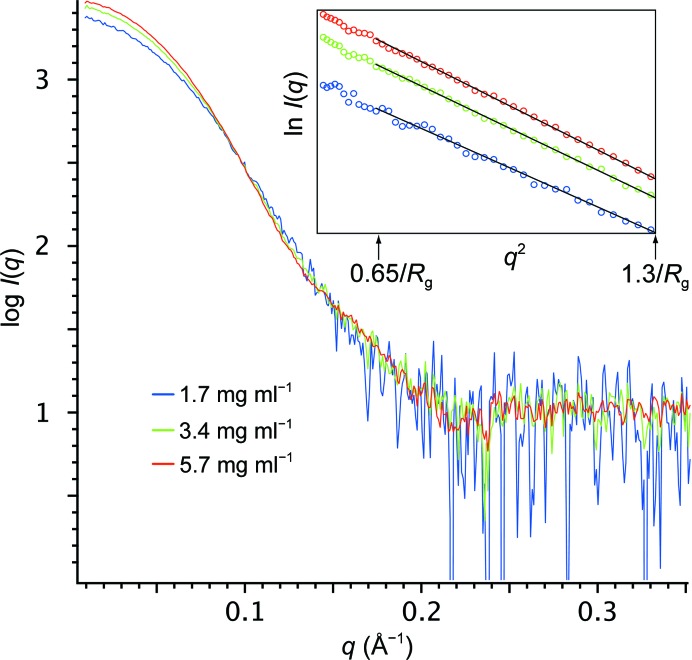
Concentration dependence detected for sample 11. Scattering profiles for the lowest (blue), middle (green) and highest (red) concentrations are shown after scaling. The increase in the *y* intercept of the data in the low-*q* region as a function of concentration reflects an increase in the size of the particle. Inset: Guinier plots for each of the three concentrations. For clarity, only the linear fits to points in Guinier region 2 are shown by black solid lines. The upper and lower limits of Guinier region 2 are noted by black arrows and labeled.

For sample number 11, several parameters were possibly (yellow) or probably (red) affected by concentration dependence. Additionally, the *I*(0) values determined from *P*(*r*) and each of the three Guinier regions were impacted by a factor of more than 12% per mg ml^−1^ with possible (yellow) or probable (red) likelihood of concentration dependence (Fig. 4 ▶, Supplementary Tables S3 and S4) even though other parameters such as *R*_g_ and *D*_max_ were not as greatly impacted (∼1%). The large increase in *I*(0), which was not reflected in the particle dimension, suggests that the average molecular weight of the particles in solution is increasing while having little effect on the *D*_max_ and *R*_g_ (§[Sec sec2.5]2.5). Similarly, the Porod volume showed an increase of 8% per mg ml^−1^ and a possible likelihood of concentration dependence. Sample 11 was one of two samples (along with sample 4) which in our previous study was shown to exist as a mixture of dimers and tetramers in solution. The concentration-dependent increase in size detected by *SAXStats* for sample 11 may therefore reflect a small shift in population from dimer to tetramer.

**Figure 5:**
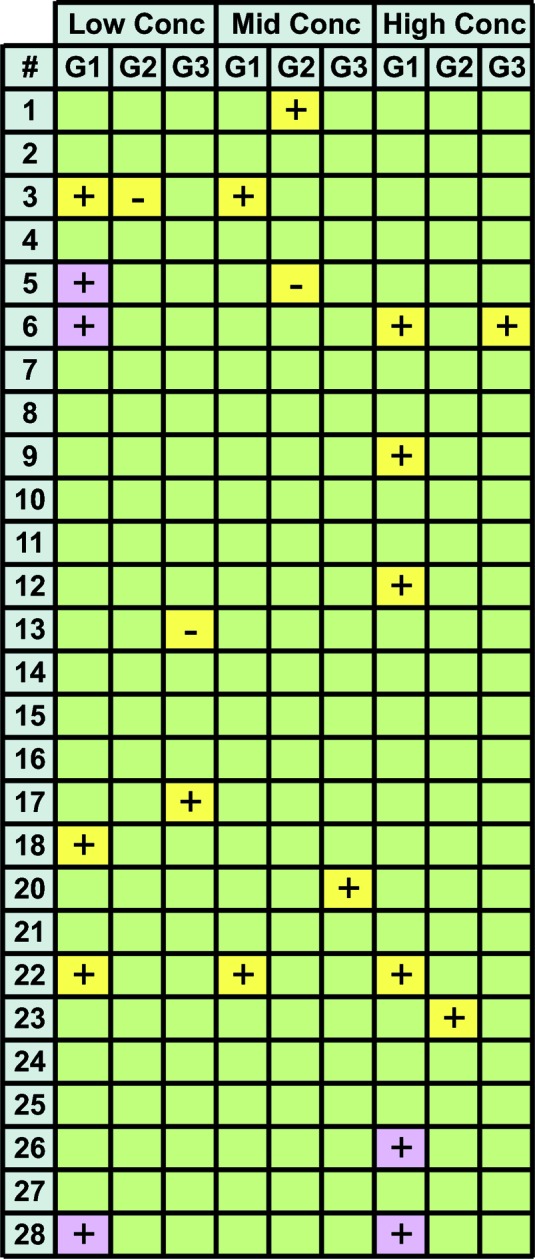
Nonlinearity evaluated for all three Guinier regions. Guinier regions that were unlikely to be (green, *p* > 0.20), possibly (yellow, 0.05 < *p* ≤ 0.20) or probably (red, *p* ≤ 0.05) nonlinear are shown for each of the three Guinier regions (G1, G2 and G3; see §[Sec sec2.5]2.5 for details). Attractive forces are denoted as positive (+) and repulsive forces as negative (−).

The evaluation of nonlinearity in each of the three Guinier regions revealed that nonlinearity was occasionally detected in Guinier region 1, the widest region encompassing data at the very lowest *q* values, and was rarely detected in Guinier regions 2 or 3 (Fig. 5 ▶). This suggests that the nonlinearity identified in Guinier region 1 is not the result of interparticle interactions but is only an artifact of slight parasitic scatter closest to the beam stop.

## 4. Discussion   

The development of the basic data-quality analysis and the *SAXStats* script has been carried out on a unique set of SAXS data where we have extremely well characterized samples (a necessary requirement before collecting SAXS data) and structural data from crystallographic, NMR or a combination of both methods (Grant *et al.*, 2011 ▶). The parameters and the molecular envelopes produced from these data in a non-automated manner have been validated by comparison to the known structures. Using *SAXStats*, we confirmed our experimental strategy to reduce radiation-damage effects and the sensitivity of our methods to detect them, and we detected small concentration dependences that had not initially been noted. These did not affect modeling or conclusions in the initial analysis, as shown by the agreement with high-resolution structural data, but serve to highlight the sensitivity of the techniques employed by the *SAXStats* script. Interestingly, we were also able to identify the case of a mixture from the initial data in sample 11 without resorting to supplementary structural knowledge. The results of the automated *SAXStats* analysis showed that most values were within 5%, and all were within 10%, of the manually determined values. Similar to the manual analysis, when compared with high-resolution structural information from X-ray crystallography or NMR, the average difference in *R*_g_ was less than 1 Å, showing that the SAXS data agreed well with the high-resolution data. The manual analysis was time-consuming and could not be performed with this level of statistical rigor in real time at the beamline. In comparison, the computational time required for *SAXStats* to be run on these samples, using a single 2.53 GHz Intel processor, was only 31 min, an average time of approximately 1 min per sample. This could be significantly reduced with more efficient coding. However, even as it stands, the reduction in processing time using *SAXStats* compared with manual analysis makes high-throughput real-time SAXS analysis a reality.

Our high-throughput protocol is designed to use the minimum necessary sample and the minimum beam time. Eight short exposures are used at each of three concentrations. Exploring the results in detail shows that a cutoff of *p* < 0.05 to identify radiation damage works well for these eight short exposures. Additionally, *p* < 0.05 is also likely to successfully identify nonlinearity in most cases since the Guinier region is likely to include a sufficient quantity of data points for well planned experiments and instrumentation setups. Using only three concentrations decreases the likelihood of identifying concentration-dependent effects, but the high number of parameters analyzed proved to be sufficient to accomplish this successfully. While using a greater number of concentrations is preferable, we have shown that it is possible to combine both the slope of the regression and the *p*-value to correctly assess the likelihood of concentration dependence and its impact on modeling and conclusions. The same analysis applied to more thorough data collection will only improve the accuracy of the results.

We note that the current analysis works most effectively for globular particles. Some parameters, such as *D*_max_ and the Porod volume, are inherently determined less accurately for highly elongated particles or for those with large disordered regions since the equations used assume globularity. Additionally, the Guinier method must be corrected for elongated or disk-shaped particles. However, given that the described method primarily detects changes in particle properties, the analyses of most parameters are still likely to be informative even in these cases. We note that the test set is small and ranges in molecular weight from ∼8.2 kDa (PDB entry 2kw9) to ∼48.5 kDa (PDB entry 3hxl). We are limited in fully testing our techniques by the availability of a robust data set for which both SAXS profiles and structural information exist. As these data become available the robustness of the analysis can be assessed and improvements, if necessary, are encouraged.

Historically, much SAXS data analysis has been performed ‘by eye’ in a highly subjective manner. In particular, the linearity of the Guinier region can be difficult to assess. The application of linear regression statistical analysis to identify radiation damage, concentration dependence and interparticle interactions provides quantitative data-quality metrics. This is particularly useful considering the growing user community for the technique. Widescale adoption of the methods employed in *SAXStats* (or similar analysis) will result in an increase in the reliability of subsequent information derived from the SAXS data by quantitatively filtering data which may mask unanticipated effects from those accurately extrapolated to a single particle.

Currently, *SAXStats* is being integrated with the existing data-collection and processing software at SSRL BL4-2. This provides near-instantaneous sample-quality feedback and thereby allows almost instant identification of sample and experimental problems which can be addressed during the data collection. We employed this procedure at BL4-2 in a testing mode. It has worked well to alert us to problematic data, demonstrating the potential to increase the success rate of SAXS experiments utilizing valuable X-ray resources and subsequently the number of scientific results produced using these resources.

While *SAXStats* is currently only employed at SSRL BL4-2, the scripts and methodology that it employs can easily be adapted to virtually any data-analysis pipeline. *SAXStats* can be used to flag problematic data that might otherwise go undetected for further manual analysis. High-throughput pipelines will be particularly benefited by the large increase in efficiency of data analysis. However, the methodology can be applied to individual systems as well. Not only does *SAXStats* enable the objective evaluation of data quality, it can also provide comparisons between varying solution conditions that may make the effects of solvent conditions on conformational changes, or oligomer organization, easier to identify. This in turn could be used to guide the sample into more desirable solution conditions, for example to increase solubility or monodispersity, or even to guide crystallization efforts. *SAXStats* does not replace expert analysis, but does flag those cases where a change in experimental design or more experience may be helpful to successfully perform the experiment and analyze the results.

## 5. Conclusions   

The methods employed in the *SAXStats* protocol along with the results of the present analysis demonstrate not only that SAXS can be performed in high throughput but that the resulting data can also be analyzed in an objective, statistically significant and efficient manner. While improvements in the efficiency and applicability of the method can certainly be made in the future, no degree of automation can remove the necessity for human intervention and analysis when it comes to drawing accurate conclusions from the data. *SAXStats* is very sensitive to the effects of concentration dependence or radiation damage; however, the influence of these effects on modelling, interpretation and the ultimate determination of biological mechanism require human insight. The simple methodology presented allows a significant amount of human intervention and subjective analysis of SAXS data to be supplemented with statistical, quantitative analyses. It provides a much-needed starting point to develop objective metrics that enable automation of data-quality assessment and opens up the technique to those more experienced in complementary data analysis.

## Supplementary Material

Supporting Information.. DOI: 10.1107/S1399004714010876/mn5059sup2.pdf

## Figures and Tables

**Table 1:** Sample details used for SAXS analysis The molecular weight of the monomeric form of each sample in given in daltons. Oligomeric states of samples were previously determined to be monomeric (M), dimeric (D), tetrameric (T) or mixtures of oligomers (Grant *et al.*, 2011 ▶). Where both crystallographic and NMR structures are available, samples are noted C and N, respectively. Full details of all of the samples are available in Grant *et al.* (2011 ▶). Note that samples 18 and 19 are identical, but are listed to be compatible with Grant *et al.* (2011 ▶). They represent structures from the same protein sample obtained through slightly differing means.

No.	Name	MW (Da)	State	PDB code
Samples with crystallographic structures				
1	Domain of unknown function	9523	M	3hz7
2	Diguanylate cyclase with PAS/PAC sensor	13611	D	3h9w
3	Nmul_A1745 protein from *Nitrosospira multiformis*	14069	T	3lmf
4	Domain of unknown function	14609	D/T	3mjq
5	Sensory box/GGDEF family protein	14779	D	3mfx
6	MucBP domain of the adhesion protein PEPE_0118	14300	M	3lyy
7	Sensory box/GGDEF-domain protein	15341	D	3lyx
8	HIT family hydrolase	17089	D	3i24
9	EAL/GGDEF-domain protein	18738	M	3icl
10	Diguanylate cyclase	20256	M	3ign
11	Putative NADPH-quinone reductase	20509	D/T	3ha2
12	MmoQ (response regulator)	32032	M	3ljx
13	Putative uncharacterized protein	48519	M	3hxl
Samples with crystallographic structures and multiple constructs				
14	Putative hydrogenase	17701	D	3lrx
15		16321	D	3lyu
16	Alr3790 protein	11670	D	3hix
17		15700	D	3hix
Samples with NMR structures				
18	MKL/myocardin-like protein 1	8276	M	2kw9
19	MKL/myocardin-like protein 1	8276	M	2kvu
20	Putative peptidoglycan-bound protein (LP*X*TG motif)	9712	M	2kvz
21	E3 ubiquitinprotein ligase Praja1	10297	M	2l0b
22	Transcription factor NF-E2 45kDa subunit	10623	M	2kz5
23	YlbL protein	10661	M	2kl1
24	Cell-surface protein	12385	M	2l0d
25	Domain of unknown function	16312	M	2kzw
26	N-terminal domain of protein PG_0361 from *Pseudomonas gingivalis*	17485	M	2kw7
Samples with both crystallographic and NMR structures				
27	GTP pyrophosphokinase	10042	D	2ko1 ^N^
		10042	D	3ibw ^C^
28	Lin0431 protein	12747	M	2kpp ^N^
		12747	M	3ld7 ^C^
