# Supplementary material for: The accurate assessment of small-angle X-ray scattering data
Source: Acta Crystallogr D Biol Crystallogr. 2015 Jan 1;71(Pt 1):45–56. doi: 10.1107/S1399004714010876 (PMC4304685; doi:10.1107/S1399004714010876)
Supplement: Supplementary file 1 [file d-71-00045-sup2.pdf]

BIOLOGICAL CRYSTALLOGRAPHY

Volume 71 (2015)

Supporting information for article:

The accurate assessment of small angle X-ray scattering data

Thomas D. Grant, Joseph R. Luft, Lester G. Carter, Tsutomu Matsui, Thomas M. Weiss, Anne Martel and Edward H. Snell

Table S1. Number of Exposures Rejected from Averaging. Column headers: “mg/ml” is the estimated concentration for each sample; “# Outliers” is the number of outliers that were rejected according to the modified z-score method described in the text; “# Damaged” is the number of exposures removed due to radiation damage; “# Averaged” is the total number of exposures that were averaged together after removal of outliers and damaged exposures.

	Low Concentration				Mid Concentration				High Concentration			
**Sample**	**mg/ml**	**# Outliers**	**# Damaged**	**# Averaged**	**mg/ml**	**# Outliers**	**# Damaged**	**# Averaged**	**mg/ml**	**# Outliers**	**# Damaged**	**# Averaged**
**1**	0.90	2	0	6	1.79	2	0	6	4.49	0	2	6
**2**	2.75	0	1	7	4.95	0	0	8	7.43	2	0	6
**3**	0.74	0	0	8	1.49	0	0	8	2.23	0	0	8
**4**	1.64	3	0	5	3.60	0	3	5	5.40	0	0	8
**5**	2.97	0	0	8	6.23	0	0	8	9.20	0	0	8
**6**	2.56	0	0	8	5.89	0	0	8	7.69	0	0	8
**7**	1.90	1	1	6	3.16	1	0	7	6.32	0	2	6
**8**	1.71	1	1	6	3.43	1	0	7	5.72	1	0	7
**9**	2.29	0	0	8	3.43	0	2	6	4.11	0	1	7
**10**	1.88	0	0	8	3.13	2	2	4	6.26	0	2	6
**11**	1.71	0	0	8	3.41	0	0	8	5.69	0	0	8
**12**	2.50	0	0	8	4.75	0	1	7	5.25	0	0	8
**13**	1.58	1	0	7	2.64	0	0	8	5.27	0	0	8
**14**	1.51	0	0	8	2.26	0	0	8	3.76	0	0	8
**15**	1.49	0	0	8	1.87	1	0	7	3.74	0	0	8
**16**	2.95	0	0	8	5.90	2	0	6	14.76	1	0	7
**17**	1.63	1	0	7	2.71	0	0	8	5.42	0	0	8
**18**	1.71	0	0	8	4.11	0	3	5	4.63	0	1	7
**20**	1.51	0	0	8	3.31	0	0	8	5.57	1	1	6
**21**	1.13	1	0	7	2.48	0	0	8	3.72	0	1	7
**22**	0.62	0	0	8	1.48	0	0	8	2.23	0	0	8
**23**	1.89	0	0	8	4.73	0	0	8	9.45	0	3	5
**24**	1.07	0	0	8	2.24	1	2	5	3.31	0	0	8
**25**	2.09	0	0	8	4.39	0	0	8	6.89	0	0	8
**26**	1.76	0	0	8	3.35	0	0	8	5.28	0	0	8
**27**	0.99	0	0	8	1.65	0	0	8	3.30	0	0	8
**28**	2.01	0	0	8	4.01	0	0	8	6.22	2	0	6

Table S2. Impact of Radiation Damage on Estimate of SAXS Parameters. The absolute value of the slope of the linear regression calculated as a function of exposure is presented in units of % change (of y-intercept of regression) per exposure. Column headers: “mg/ml” is the estimated concentration of each sample in mg/ml; “χ” is the similarity of scattering profiles between each exposure compared to the first exposure; “R_g” is calculated using Guinier region 3; “R_g-*P(r)*” is calculated via the *P(r)* distribution; “D_{max}” is the maximum particle dimension; “*I(0)*” is the forward scattering extrapolated from Guinier region 3.

	**Low Concentration**						**Mid Concentration**						**High Concentration**					
**Sample**	**mg/ml**	**χ**	**R_g**	**R_g-P(r)**	**Dmax**	**I(0)**	**mg/ml**	**χ**	**R_g**	**R_g-P(r)**	**Dmax**	**I(0)**	**mg/ml**	**χ**	**R_g**	**R_g-P(r)**	**Dmax**	**I(0)**
**1**	0.90	0.12	1.88	0.63	1.87	1.63	1.79	1.47	0.09	0.22	1.63	0.28	4.49	2.37	0.86	0.23	0.35	1.25
**2**	2.75	0.40	1.07	0.19	0.50	1.28	4.95	0.26	0.44	0.13	0.21	0.56	7.43	1.28	0.11	0.05	0.24	0.21
**3**	0.74	0.40	0.54	0.15	0.46	0.45	1.49	0.35	0.58	0.10	1.18	0.51	2.23	0.06	0.39	0.13	0.63	0.25
**4**	1.64	0.11	0.15	0.01	0.20	0.18	3.60	2.11	0.11	0.05	0.02	0.01	5.40	0.55	0.14	0.06	0.00	0.20
**5**	2.97	0.60	0.20	0.13	0.74	0.28	6.23	0.55	0.07	0.03	0.69	0.08	9.20	0.52	0.13	0.02	0.05	0.02
**6**	2.56	0.78	0.55	0.07	0.31	0.35	5.89	0.50	1.08	0.07	0.09	0.75	7.69	0.11	0.18	0.02	0.04	0.14
**7**	1.90	0.38	0.35	0.29	0.32	0.47	3.16	0.55	0.07	0.03	0.41	0.06	6.32	3.15	0.05	0.26	0.30	0.45
**8**	1.71	0.91	0.50	0.15	0.39	0.35	3.43	0.26	0.28	0.05	0.79	0.14	5.72	0.27	0.00	0.06	0.17	0.20
**9**	2.29	0.77	0.04	0.37	0.58	0.09	3.43	1.19	0.60	0.02	0.69	0.25	4.11	0.11	0.29	0.34	1.24	0.02
**10**	1.88	0.24	1.30	0.12	0.24	0.63	3.13	0.85	0.37	0.17	0.34	0.31	6.26	1.43	0.01	0.10	0.22	0.16
**11**	1.71	1.00	0.51	0.11	0.07	0.66	3.41	0.67	0.36	0.08	0.21	0.24	5.69	0.32	0.23	0.00	0.16	0.19
**12**	5.25	1.58	1.01	5.01	9.94	2.12	4.75	0.06	0.55	0.16	0.42	0.31	2.50	0.27	0.24	0.17	0.08	0.35
**13**	1.58	0.66	1.33	0.13	0.57	0.54	2.64	0.11	0.47	0.03	0.27	0.29	5.27	0.21	0.05	0.17	0.56	0.22
**14**	1.51	0.47	0.07	0.07	0.32	0.20	2.26	0.83	0.49	0.03	0.23	0.44	3.76	0.16	0.65	0.15	0.10	0.64
**15**	1.49	0.72	0.52	0.06	1.03	0.23	1.87	0.88	0.15	0.18	0.22	0.06	3.74	0.50	0.72	0.10	0.13	0.89
**16**	2.95	0.21	1.03	0.10	0.12	0.90	5.90	0.37	0.64	0.11	0.39	0.54	14.76	1.40	0.33	0.03	0.23	0.47
**17**	1.63	0.20	0.93	0.14	0.43	0.59	2.71	0.45	0.43	0.12	0.01	0.17	5.42	0.06	0.72	0.41	1.08	0.36
**18**	1.71	0.49	0.67	0.10	0.22	0.13	4.11	0.11	0.26	0.40	0.80	0.35	4.63	1.62	0.31	0.02	0.11	0.32
**20**	1.51	0.54	2.10	0.62	0.63	1.13	3.31	0.45	0.28	0.28	0.56	0.47	5.57	0.08	1.11	0.29	0.48	0.77
**21**	1.13	0.01	0.25	0.48	0.13	0.30	2.48	0.49	0.30	0.39	0.61	0.34	3.72	0.99	0.65	0.61	1.83	0.81
**22**	0.62	0.87	3.19	1.24	1.75	2.30	1.48	0.43	0.85	0.38	0.45	0.65	2.23	0.11	0.69	0.17	0.19	0.35
**23**	1.89	0.76	2.51	0.49	0.36	1.66	4.73	0.31	0.49	0.19	0.36	0.62	9.45	0.30	0.63	0.22	0.42	0.62
**24**	1.07	0.73	0.68	0.19	0.29	0.57	2.24	0.88	0.29	0.04	0.40	0.03	3.31	1.05	1.13	0.03	0.15	0.82
**25**	2.09	0.29	0.53	0.56	1.35	0.50	4.39	0.34	0.44	0.65	1.39	0.49	6.89	0.60	0.07	0.03	0.36	0.13
**26**	1.76	0.72	0.92	0.49	0.53	0.80	3.35	0.00	0.27	0.11	0.09	0.24	5.28	0.45	0.19	0.17	0.48	0.14
**27**	0.99	1.10	1.68	0.22	0.58	0.68	1.65	1.00	1.79	0.12	1.07	1.36	3.30	0.42	0.27	0.04	0.49	0.58
**28**	2.01	1.76	0.36	0.23	0.24	0.25	4.01	1.82	0.25	0.18	0.56	0.36	6.22	0.20	0.25	0.24	0.66	0.36

Table S3. Likelihood of concentration dependence expressed as p-value for each of the 28 samples examined. “-1”, “-2”, “-3”, and “-P(r)” refer to parameters evaluated using Guinier region 1, Guinier region 2, Guinier region 3, and the pair distribution function, respectively. “*I(0)/c”* refers to the forward scattering value determined *after* scaling, and thus is independent of concentration. “Porod” refers to the molecular weight calculated from the Porod volume.

Sample ID	Rg-1	Rg-2	Rg-3	I(0)/c-1	I(0)/c-2	I(0)/c-3	Rg-P(r)	I(0)-P(r)	Dmax	Porod
1	>0.2	>0.2	>0.2	>0.2	>0.2	>0.2	>0.2	>0.2	0.1	>0.2
2	0.2	>0.2	>0.2	>0.2	>0.2	0.005	0.2	>0.2	0.01	0.2
3	>0.2	>0.2	>0.2	0.2	>0.2	>0.2	>0.2	0.1	>0.2	0.2
4	0.2	0.2	0.2	0.2	0.2	0.2	0.05	0.05	>0.2	0.2
5	0.05	0.02	>0.2	0.02	0.05	0.2	0.2	0.1	0.2	>0.2
6	0.2	>0.2	>0.2	0.1	0.1	0.05	0.2	0.1	0.1	>0.2
7	>0.2	0.2	0.1	0.1	0.005	0.005	0.05	0.1	>0.2	0.05
8	>0.2	>0.2	>0.2	>0.2	>0.2	>0.2	>0.2	>0.2	0.2	>0.2
9	>0.2	>0.2	>0.2	0.001	0.02	0.1	>0.2	0.2	>0.2	0.2
10	>0.2	>0.2	>0.2	0.2	0.2	>0.2	0.2	0.2	>0.2	>0.2
11	0.2	0.2	0.01	0.2	0.2	0.1	>0.2	0.2	>0.2	0.1
12	>0.2	>0.2	>0.2	>0.2	>0.2	>0.2	0.1	>0.2	>0.2	>0.2
13	>0.2	>0.2	>0.2	>0.2	>0.2	>0.2	0.2	0.05	0.2	>0.2
14	>0.2	>0.2	>0.2	0.2	0.2	>0.2	>0.2	0.2	>0.2	0.2
15	0.05	0.02	>0.2	0.2	0.2	0.02	0.05	0.2	>0.2	>0.2
16	0.02	0.05	>0.2	0.1	0.2	0.05	0.1	0.2	0.1	0.2
17	>0.2	>0.2	>0.2	0.2	>0.2	>0.2	0.2	>0.2	0.2	>0.2
18	>0.2	0.2	>0.2	>0.2	0.005	>0.2	>0.2	>0.2	>0.2	>0.2
20	0.2	>0.2	>0.2	0.05	0.05	0.05	0.1	0.005	>0.2	0.2
21	>0.2	0.2	>0.2	>0.2	0.2	>0.2	>0.2	>0.2	>0.2	>0.2
22	>0.2	>0.2	>0.2	>0.2	>0.2	>0.2	>0.2	>0.2	>0.2	>0.2
23	>0.2	>0.2	>0.2	0.2	0.2	>0.2	0.02	0.01	0.05	0.2
24	0.1	>0.2	>0.2	0.01	0.2	0.2	>0.2	0.2	>0.2	>0.2
25	>0.2	>0.2	>0.2	>0.2	>0.2	>0.2	>0.2	>0.2	>0.2	>0.2
26	0.2	>0.2	>0.2	0.1	0.1	>0.2	0.2	0.1	0.2	>0.2
27	0.2	>0.2	>0.2	0.2	0.2	>0.2	0.1	0.2	>0.2	>0.2
28	0.2	0.2	0.2	0.2	0.2	0.1	0.2	0.1	0.2	>0.2

Table S4. Impact of Concentration Dependence on Estimate of SAXS Parameters. The absolute value of the slope of the linear regression calculated as a function of concentration is presented in units of % change (of y-intercept of regression) per mg/ml. Column headers explained in caption of Table S3.

Sample ID	Rg-1	Rg-2	Rg-3	I0/c-1	I0/c-2	I0/c-3	Pr-Rg	Pr-I0	Dmax	Porod-MW	Average	Median
**1**	3.23	3.16	3.28	3.74	3.69	3.87	3.33	3.71	5.53	5.07	**3.86**	**3.70**
**2**	0.83	0.59	0.25	0.31	0.51	0.85	0.63	0.51	1.41	2.87	**0.88**	**0.61**
**3**	0.89	2.47	2.80	1.22	2.48	2.99	0.49	1.08	3.64	9.20	**2.73**	**2.48**
**4**	0.79	0.61	0.34	0.58	0.48	0.25	1.39	0.69	12.53	1.48	**1.91**	**0.65**
**5**	0.88	0.78	0.30	0.97	0.88	0.45	0.50	0.68	1.35	0.56	**0.74**	**0.73**
**6**	1.31	0.73	0.13	1.68	1.35	0.94	1.61	1.79	3.16	2.86	**1.56**	**1.48**
**7**	0.51	0.65	0.90	0.48	0.59	0.78	0.65	0.51	0.53	1.40	**0.70**	**0.62**
**8**	0.24	0.00	0.14	0.02	0.16	0.02	0.63	0.22	4.75	0.44	**0.66**	**0.19**
**9**	1.73	2.04	2.53	2.66	2.93	3.36	5.06	3.48	26.47	9.12	**5.94**	**3.15**
**10**	0.66	1.01	2.48	1.32	1.58	2.81	0.55	1.26	0.75	4.12	**1.65**	**1.29**
**11**	1.14	1.14	1.78	12.21	12.21	13.37	1.01	12.13	0.47	7.59	**6.31**	**4.69**
**12**	0.39	0.31	0.76	0.51	0.42	0.82	0.48	0.46	0.77	1.13	**0.61**	**0.50**
**13**	0.00	0.00	0.45	0.25	0.25	0.08	0.35	0.40	0.22	0.53	**0.25**	**0.25**
**14**	0.33	0.47	0.86	1.57	1.70	2.05	0.05	1.43	0.14	1.83	**1.04**	**1.15**
**15**	0.66	0.70	0.41	1.15	1.09	2.14	0.31	1.42	0.72	3.19	**1.18**	**0.91**
**16**	0.33	0.27	0.27	1.12	1.09	1.08	0.38	1.15	0.91	2.07	**0.87**	**1.00**
**17**	0.81	0.30	1.34	0.53	0.18	1.02	0.54	0.38	1.50	4.97	**1.16**	**0.68**
**18**	0.12	1.06	0.24	0.30	0.94	0.06	0.06	0.30	0.67	2.91	**0.67**	**0.30**
**20**	4.21	0.92	0.05	4.34	2.11	1.15	2.34	2.87	2.50	7.36	**2.79**	**2.42**
**21**	0.70	0.99	0.49	0.07	0.94	0.54	0.61	0.21	1.10	3.97	**0.96**	**0.66**
**22**	2.14	2.49	0.76	1.60	1.96	0.52	1.08	1.27	0.55	8.98	**2.14**	**1.44**
**23**	0.87	1.28	0.00	0.90	1.17	0.03	0.72	0.75	2.09	4.06	**1.19**	**0.89**
**24**	5.99	5.61	4.40	6.56	6.27	5.34	5.55	5.92	3.54	11.34	**6.05**	**5.77**
**25**	1.36	0.15	0.40	0.95	0.02	0.25	0.33	0.13	0.15	0.37	**0.41**	**0.29**
**26**	1.02	0.35	0.36	1.23	0.73	0.71	1.09	1.09	7.88	3.58	**1.80**	**1.06**
**27**	5.13	6.13	6.38	9.19	10.05	10.37	5.15	8.94	8.86	13.08	**8.33**	**8.90**
**28**	9.51	2.62	3.32	5.67	1.93	2.39	8.19	3.45	120.78	1.28	**15.91**	**3.39**
**Average**	**1.40**	**1.32**	**1.23**	**2.13**	**2.15**	**2.15**	**1.34**	**2.03**	**3.55**	**4.39**	**2.17**	**1.76**
**Median**	**0.85**	**0.76**	**0.47**	**1.14**	**1.09**	**0.90**	**0.63**	**1.09**	**1.38**	**3.39**	**1.18**	**0.95**
